# Impact of Neuroendocrine Differentiation (NED) on Enzalutamide and Abiraterone Efficacy in Metastatic Castration-Resistant Prostate Cancer (mCRPC): A Retrospective Analysis

**DOI:** 10.3390/cells13161396

**Published:** 2024-08-22

**Authors:** Giovanni Farinea, Mariangela Calabrese, Federica Carfì, Isabella Saporita, Stefano Poletto, Marco Donatello Delcuratolo, Fabio Turco, Marco Audisio, Francesco Rosario Di Stefano, Marcello Tucci, Consuelo Buttigliero

**Affiliations:** 1 Department of Oncology, San Luigi Gonzaga University Hospital, University of Turin, 10043 Orbassano, Italy; mariangela.calabrese@unito.it (M.C.); federicamaria.carfi@unito.it (F.C.); isabella.saporita@unito.it (I.S.); stefano.poletto@unito.it (S.P.); donatello.m.delcuratolo@gmail.com (M.D.D.); marco.audisio@unito.it (M.A.); rosario-distefano@virgilio.it (F.R.D.S.); consuelo.buttigliero@unito.it (C.B.); 2 Oncology Institute of Southern Switzerland, 6500 Bellinzona, Switzerland; fabio.turco@eoc.ch; 3 Department of Medical Oncology, Cardinal Massaia Hospital, 14100 Asti, Italy; marcello.tucci@gmail.com

**Keywords:** prostate cancer, androgen receptor pathway inhibitor, neuroendocrine tumors, castration-resistant prostate cancer

## Abstract

Neuroendocrine differentiation (NED) represents a possible androgen receptor pathway inhibitors (ARPI) resistance mechanism in metastatic castration resistance prostate cancer (mCRPC). As mCRPC with NED has been excluded from clinical trials evaluating ARPI efficacy, this study investigates the prognostic impact of NED in mCRPC patients treated with ARPIs. **Methods:** We retrospectively analyzed 327 mCRPC patient data treated with Enzalutamide or Abiraterone in the first and second or successive lines of treatment. NED was assessed using prostate biopsy samples through immunohistochemical staining. **Results:** NED was confirmed in 32/327 (9.8%) mCRPC patients. In the overall population, mCRPC with NED showed worse PFS (4.38 vs. 11.48 months HR 2.505 [1.71–3.68] *p* < 0.05), disease control rate (DCR), and PSA response. In the first line setting, mCRPC with NED demonstrated worse PFS (8.5 vs. 14.9 months HR 2.13 [1.18–3.88], *p* < 0.05). Similarly, in the second or successive lines, mCRPC with NED showed worse PFS (4.0 vs. 7.5 months HR 2.43 [1.45–4.05] *p* < 0.05), DCR, PSA response and OS (12.53 vs. 18.03 months HR 1.86 [1.12–3.10] *p* < 0.05). The adverse impact of NED on PFS was consistence across all subgroups; we also noted a trend of worse PFS in patients with high vs. low NED. **Conclusions:** In our study, mCRPC with NED treated with Enzalutamide or Abiraterone showed worse clinical outcomes. NED assessment should be considered to optimize treatment decisions in the mCRPC setting.

## 1. Introduction

Prostate cancer (PC) is the most common malignancy in men in the United States and Europe, representing the second leading cause of cancer-associated death [1].

At diagnosis PC is an androgen-dependent disease, relying on ligand-mediated signaling via the androgen receptor (AR) for tumor growth. Therefore, the inhibition of the AR pathway represents the mainstay of advanced PC therapy [2]. However, under treatment pressure, PC develops a variety of resistance mechanisms, ultimately progressing to metastatic castration-resistant prostate cancer (mCRPC) in nearly all patients [3,4].

As the majority of mCRPC cases retain a high dependency on AR signaling, new-generation hormonal agents, through deeper AR pathway inhibition, effectively suppress tumor growth even in the castration-resistant setting.

While the efficacy of androgen receptor pathway inhibitors (ARPIs) has been demonstrated in many clinical trials, approximately 25–35% of mCRPC patients treated with abiraterone or enzalutamide exhibit primary resistance to these agents [5,6,7,8].

Primary and acquired ARPI resistance depend on both AR-dependent or AR-independent mechanisms, including the up-regulation of systemic and intratumoral androgen biosynthesis, AR gene mutations and amplifications, alteration of pathways implicated in crosstalk with AR signaling, glucocorticoid receptor overexpression, immune system deregulation, and neuroendocrine differentiation (NED) [8,9].

NED represents a well-recognized mechanism of AR-independent resistance to hormonal therapy [10,11].

NED in PC represents a complex, androgen-independent phenotype that can arise either de novo or in advanced stages of cancer progression as a mechanism of treatment resistance and is driven by gene alterations including loss of tumor suppressors PTEN, RB1, TP53 [12,13].

Two main hypotheses, only partially contradictory, explain the origin of PC with NED: the hierarchical model and the dynamic trans-differentiation model.

The first model suggests that resident neuroendocrine cells [14], before initiation of endocrine therapies, represent a minority of cells as they are outgrown by the AR-positive adenocarcinoma cells. However, after AR inhibition, their AR independence is a major growth advantage [15].

The second model assumes that tumor cells can acquire phenotypic characteristics of a cell lineage whose survival does not depend on the drug target [16,17].

This trans-differentiation model suggests that adenocarcinoma cells undergo lineage switching to the neuroendocrine lineage via genetic and epigenetic dysregulation relinquishing their dependence on the AR pathway and activating alternative mitogenic pathways, epithelial–mesenchymal transition, and stem cell programs [10,11,18].

Morphologically, PC cells exhibiting NED share features with other high-grade neuroendocrine cancers, including the presence of small cells with “salt and pepper” chromatin, positive staining for neuroendocrine immunohistochemical markers such as neuron-specific enolase, synaptophysin (Syn), chromogranin (CgA), or CD56 and a high mitotic rate [19,20].

Clinically, the presence of neuroendocrine cells is associated with an aggressive disease phenotype, marked by rapid tumor progression, visceral metastasis development, and resistance to conventional therapies [12,21]. The presence of NED can be suspected in case of radiologic progression with visceral and/or lytic bone metastases despite low or moderately rising PSA levels [20].

NED may be detected at the time of initial diagnosis in approximately 2% of prostate cancer cases (“de novo”), or it may arise during hormonal treatment in 20–25% of cases [22,23,24].

As NED can act as a potential resistance mechanism to conventional hormonal agents, patients with mCRPC exhibiting NED were generally excluded from trials evaluating the efficacy of ARPIs [5,6,7,8].

Consequently, the prognostic significance of neuroendocrine markers in mCRPC patients treated with ARPIs remains uncertain.

Recent studies show a correlation between circulating markers of NED and unfavorable clinical outcomes in patients with mCRPC [25,26,27,28], however, these serum markers may be influenced by drug administration or the presence of concurrent comorbidities [29,30,31].

In comparison, evaluating the neuroendocrine status directly in tumor tissue is not influenced by other conditions, making it more reliable and suitable for clinical practice.

**Figure 1:**
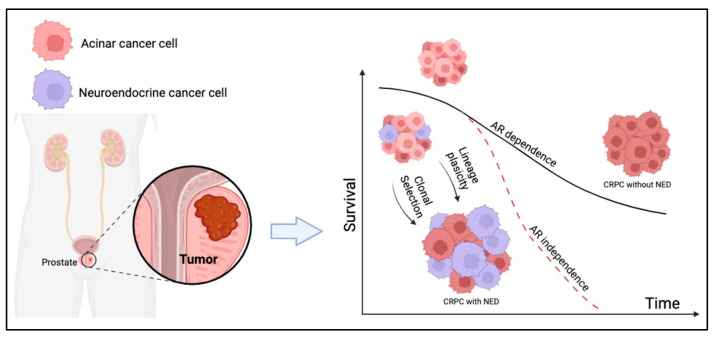
Neuroendocrine differentiation (NED) is a dynamic process during prostate cancer progression and represents a mechanism of androgen receptor pathway inhibitors (ARPIs) resistance in metastatic castration-resistant prostate cancer (mCRPC).

This study investigates the prognostic impact of NED in patients with mCRPC undergoing treatment with Enzalutamide and Abiraterone (Figure 1).

This figure graphically represents the conclusions of our study, which, through NED evaluation in tumor tissue, we showed that the presence of neuroendocrine clones leads to AR-independent tumor growth and reduced survival in mCRPC patients with NED (red line in Kaplan–Meier survival curve) compared with mCRPC patients without NED (black line in Kaplan–Meier survival curve).

## 2. Materials and Methods

### 2.1. Study Population

This retrospective study reviewed clinical records of all mCRPC patients treated with Enzalutamide or Abiraterone Acetate at our Medical Oncology division from 2010 to 2023.

Patients received treatment following the standard schedule: Enzalutamide (160 mg/die) or Abiraterone Acetate (1000 mg/die in combination with prednisone 10 mg/die).

Baseline data collected for each patient are presented in Table 1. NED was defined as the presence of tumor cells with neuroendocrine features (identified morphologically and by immunohistochemical positivity for CgA and/or Syn), in tumor tissue. Tumor tissue of primary tumor was obtained at diagnosis (prostate biopsy or prostatectomy) or after first-line hormonal therapy but before ARPI or Docetaxel treatment in mCRPC setting.

Patients were categorized according to the presence and proportion of neuroendocrine cells. The extent of NED was categorized into two groups: low NED (1–49% of neuroendocrine tumor cells) and high NED (>50% of neuroendocrine tumor cells).

### 2.2. Treatment Outcomes Measures

Outcome measures included progression-free survival (PFS), overall survival (OS), radiologic response (RECIST 1.1 and/or Prostate Cancer Clinical Trials Working Group 3 [PCWG3] criteria), and best PSA response. Radiologic tumor response were evaluated through CT scans and bone scintigraphy every 3 months or as clinically indicated.

PFS was defined as the interval from the start of ARPIs to radiologic progression, death, or last follow-up for live patients. OS was the time from the start of ARPIs to death or last follow-up for alive.

The radiologic disease control rate (DCR) was defined as the presence of stable disease (SD), partial response (PR), and complete (CR) according to RECIST 1.1 and/or PCWG3 criteria. The radiologic overall response rate (ORR) was defined as the presence of PR or CR according to RECIST 1.1 and/or PCWG3 criteria.

The best PSA response was defined as the greatest reduction from baseline and was classified as progression, 0–50% reduction, and >50% reduction.

Treatment outcomes were analyzed in the overall population and separately based on previous systemic treatment in mCRPC setting (pre- vs. post-DOCETAXEL).

### 2.3. Statistical Analysis

PFS and OS were calculated using the Kaplan–Meier method, and survival relative risk was assessed using Cox regression method. The median follow-up was determined using the Schemper method. Statistical significance of between-group comparisons was evaluated using the log-rank and chi-squared tests, with *p*-values < 0.05 considered significant.

The analyses were performed with the IBM SPSS Statistic program (version 25.0) and with GraphPad Prism (version 10.0).

## 3. Results

### 3.1. Baseline Characteristics

Baseline characteristics of the study population are summarized in Table 1.

Three-hundred and twenty-seven mCRPC patients treated with Abiraterone or Enzalutamide were included in the analysis, with a median follow-up of 54.51 months (IC 95% 48.13–60.88).

The median age was 66 years (range 47–87 years). At the initiation of ARPIs, 313 patients (95.3%) had Eastern Cooperative Oncology Group Performance Status (ECOG PS) of 0–1 and 14 (4.7%) had an ECOG PS = 2.

All participants had advanced PC, with metastases present at initial diagnosis in 153 cases (46.8%).

Two hundred and sixty-two patients (80.4%) had bone metastases, two hundred (61.2%) had node metastases and thirty-nine (12.1%) had visceral metastases.

ARPIs were administered as first-line treatment in 199 (60.8%) patients and as second or successive line (post-docetaxel) in 128 patients (39.2%). All patients previously treated in the mCRPC setting had received taxane-based chemotherapy as first-line treatment.

Baseline median PSA was 29.1 ng/mL (interquartile range [IQR] 0.1–139.3). Baseline median hemoglobin (Hb), alkaline phosphatase (ALP), and lactic dehydrogenase (LDH) were 12.8 g/dl (IQR: 11–14.6 g/dL), 91 Ul/l (IQR: 35–147 UI/L) and 235 UI/L (IQR: 153–317 UI/L), respectively.

A total of 96 cases of prostate cancer (34.3%) were classified as International Society of Urological Pathology (ISUP) grade 4/5, while 184 cases (65.7%) were classified as ISUP 1–3.

Neuroendocrine differentiated cells were detected in 32 patients (9.8%): in 29 cases at diagnosis and in 3 cases after androgen deprivation therapy initiation (before ARPIs or Docetaxel treatment mCRPC setting). All tumor samples used for NED assessment were collected from primary tumors (prostatectomy or prostate biopsy). In total, 20 patients (62.5%) had a low neuroendocrine proportion, and 12 patients (37.5%) had a high neuroendocrine proportion, respectively.

Treatment outcomes were analyzed according to previous systemic treatment in the mCRPC setting.

In patients treated with first-line ARPIs, the PSA response rate was 73.1% (141/193), the overall DCR was 64.0% (108,167), the median PFS was 14.51 months (95% CI 12.44–16.56), and the median OS was 29.28 months (95% CI 23.37–35.16) (Table S1).

In patients treated with ARPIs as the second or subsequent line, the PSA response rate was 52.2% (59/113), the overall DCR was 45.7% (43/94), the median PFS was 6.38 months (95% CI 4.97–7.79), and the median OS was 17.50 months (95% CI 14.18–20.81) (Table S1).

### 3.2. Baseline Characteristics Comparison between mCRPC With and Without NED

As depicted in Table 1, we initially compared the baseline characteristics of mCRPC patients with and without NED.

In the overall cohort, mCRPC with NED exhibited a higher incidence of metastasis at diagnosis (68.8 vs. 44.4% *p* < 0.05) and visceral metastases (28.1 vs. 10.3% *p* < 0.05) compared to mCRPC without NED. Furthermore, mCRPC with NED were more frequently treated with ARPIs in second or subsequent lines of treatment (56.3 vs. 37.3% *p* < 0.05). There was no significant difference observed in PSA levels between mCRPC patients with NED and those without NED.

### 3.3. Treatment Outcomes Comparison between mCRPC With and Without NED

Treatment Outcomes are summarized in Table 2.

**Figure 2:**
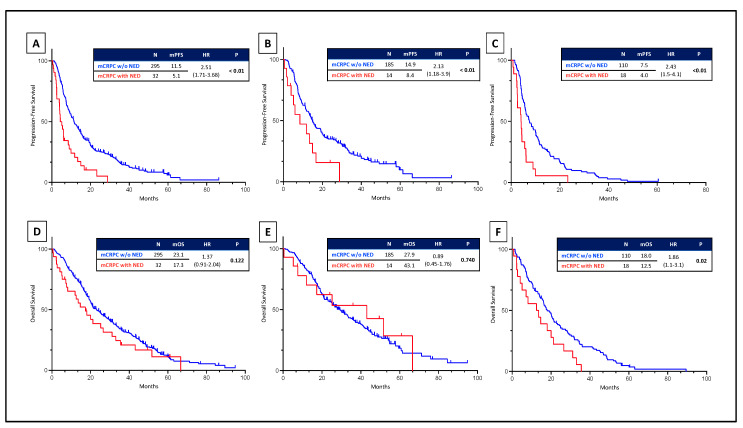
Kaplan–Meier curves representing progression-free survival (PFS) and overall survival (OS) in metastatic castration-resistant prostate cancer (mCRPC) with or without neuroendocrine differentiation (NED) treated with Enzalutamide or Abiraterone. PFS analysis in overall population (**A**), pre-DOCETAXEL (**B**), and post-DOCETAXEL (**C**). OS Analysis in overall population (**D**), pre-DOCETAXEL (**E**), and post-DOCETAXEL (**F**).

**Figure 3:**
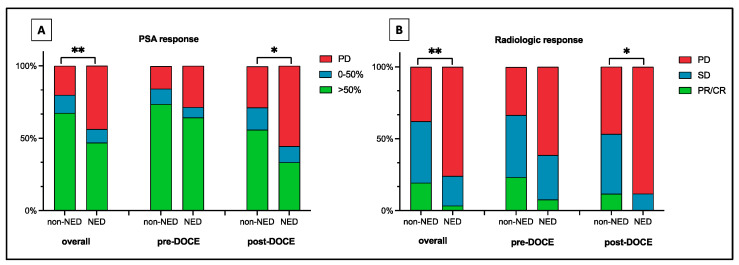
Histograms showing (**A**) PSA and (**B**) radiographic response of patients with castration-resistant prostate cancer (mCRPC) with and without neuroendocrine differentiation (NED) in the overall cohort. Pre-DOCETAXEL (pre-DOCE) and post-DOCETAXEL (post-DOCE) setting. In patients with mCRPC treated with Enzalutamide or Abiraterone in pre-DOCE setting the presence of NED was not associated with worse PSA (*p* = 0.21) and radiologic (*p* = 0.067) response. Contrarily, in patients with mCRPC treated with Enzalutamide or Abiraterone in post-DOCE setting the presence of NED was associated with worse PSA (*p* = 0.024) and radiologic (*p* = 0.02) response. (** *p* < 0.01; * *p* < 0.05).

In the overall cohort, mCRPC with NED showed worse PFS (4.38 vs. 11.48 months HR 2.51 [1.71–3.68] *p* < 0.05), DCR (24.10 vs. 62.10% OR 0.20 [IC95 0.47–0.1] *p* < 0.05) and PSA response and a trend towards worse OS (17.3 vs. 23.1 months HR 1.37 [0.92–2.04] *p* = 0.122) (Figure 2 and Figure 3).

In the pre-DOCETAXEL group, mCRPC patients with NED treated with ARPI showed worse PFS (8.49 vs. 14.93 months HR 2.11 [1.16–3.82], *p* < 0.05) but no significant difference in DCR (38.50 vs. 66.50% OR 0.32 [IC95 1.02–0.10] *p* = 0.067, PSA response and OS (43.10 vs. 29.20 months HR 0.88 [0.45–1.75] *p* = 0.732) (Figure 2 and Figure 3).

In the post-DOCETAXEL group, mCRPC patients with NED treated with ARPIs showed worse PFS (4.01 vs. 7.47 months HR 2.43 [1.45–4.05], *p* < 0.05), DCR (11.80 vs. 53.20 OR 0.12 [0.03–0.55] *p* < 0.05), PSA response and OS (12.53 vs. 18.03 months HR 1.86 [1.11–3.10] *p* < 0.05) (Figure 2 and Figure 3).

Finally, subgroup analyses revealed that mCRPC with high NED experience shorter PFS compared to mCRPC with a low NED, both in the overall population (2.5 vs. 6.0 months, *p* = 0.028) and in the pre-DOCETAXEL group (13.4 vs. 1.7 months, *p* = 0.01) (Figure S1).

### 3.4. Univariate/Multivariate and Subgroup Analysis

In univariate and multivariate analyses, the presence of NED was as significantly associated with poorer PFS (HR 2.13 [1.34–3.39], *p* < 0.05) as shown in Tables S2–S4. Furthermore, the impact of NED on PFS remained consistent across all subgroups, as shown in Figure S2A,B.

Interestingly, in the univariate analysis, the presence of NED was associated with worse OS in the post-DOCETAXEL (HR 1.86 [1.15–3.10] *p* = 0.017) but not in the pre-docetaxel setting (HR 0.89 [0.45–1.76] *p* = 0.741) (Tables S2 and S3).

## 4. Discussion

The advent of ARPIs signifies a crucial advancement in the treatment landscape of mCRPC. Nevertheless, a subset of patients fails to respond or experience rapid disease progression reflecting that mCRPC encompasses a wide spectrum of diseases ranging from slowly progressive tumors with elevated ARPI sensitivity to highly aggressive variants of prostate cancer characterized by rapid tumor and low response to hormonal agents [32,33].

Although extensive research has investigated the main mechanisms of resistance to ARPIs, the lack of reliable predictive biomarkers remains a significant challenge in tailoring treatment strategies to individual patients [33].

Aggressive variant prostate cancers (AVPCs) are characterized by distinct clinical features like rapid tumor growth, presence of visceral and lytic bone metastasis, prostate-specific membrane antigen (PSMA)-low and fluorodeoxyglucose (FDG)-high uptake on PET scan, limited response to androgen deprivation therapy and neuroendocrine differentiation [9,13].

Neuroendocrine prostate cancer cells express neuroendocrine markers (Syn and CgA) and genomic analysis reveals frequent biallelic inactivation of TP53, RB1, and PTEN. Interestingly, loss of tumor suppressors is associated with lineage plasticity, AR-independent tumor growth [11,13,34,35,36], and ARPI resistance in pre-clinical models [37].

Beyond these genomic alterations, several studies showed that the expression and activity of several proteins involved in transcriptional and epigenetic regulation (such as induction of MYCN, ASCL1, FOXA2, SOX2, EZH2, PHF8 expression as well as down-regulation of FOXA1 or NKX3–1) are often altered in neuroendocrine cells and can lead to anti-androgen resistance [32,38,39].

As the acquisition of a neuroendocrine phenotype grants tumor cells independence from AR stimulation, individuals with mCRPC exhibiting NED are usually excluded from clinical trials [5,6,7,8].

Treatment for neuroendocrine PC is largely based on efficacy data for small cell lung cancer and platinoids combined with etoposide/taxanes are the most used and internationally endorsed regimens. However, the prognosis still remains poor [40,41,42]. Despite the novel potential therapies that are currently under development, the best treatment option for this subgroup of PC is unclear [35,43,44,45].

Serum CgA and other circulating neuroendocrine markers have been associated with unfavorable outcomes in mCRPC patients [25,26,27,28]. However, their interpretation may be confounded by other medications and concurrent diseases [29,30,31].

In addition, despite the promising performance of liquid biopsy in diagnosing and monitoring neuroendocrine clones during tumor progression, its clinical role remains undetermined [46,47].

Conversely, tissue assessment of NED is unaffected by external factors, making it easily implementable in clinical practice and enabling quantification of the neuroendocrine proportion [22,48].

This study aims to assess the prevalence, clinical characteristics, and prognostic impact of NED among patients with mCRPC treated with ARPIs.

Previous studies have reported NED in approximately 2% of PC cases at diagnosis, with the detection rate increasing during cancer progression [49,50]. In our cohort of mCRPC patients, we identified a higher frequency (8–9%) of neuroendocrine cells detection at initial diagnosis; which could rise up to 11–40% following mCRPC transformation as reported by previous findings [48,51,52].

Our analysis shows that NED not only represents an independent adverse prognostic factor for ARPI-treated mCRPC but is correlated with other unfavorable prognostic factors, such as visceral metastases.

In our study, we focused on how the presence of NED impacts the efficacy of ARPIs in mCRPC settings.

Clinical outcomes were analyzed in the overall cohort and separately according to prior chemotherapy treatment, revealing a negative prognostic impact of the presence of NED in all subgroups.

Consistently, previous analyses showed a detrimental impact of NED in Asian mCRPC patients treated with first-line Abiraterone [48]. In their study, Xu and colleagues retrospectively analyzed the impact of NED, assessed through tumor biopsy samples at the time of mCRPC diagnosis, on the efficacy of abiraterone or docetaxel as first-line treatment in 262 mCRPC. NED was detected in 100/262 (38.2%) patients, among them 76/100 with a limited neuroendocrine component (<10% of tumor cells) and 24/100 with an elevated neuroendocrine component (≥10% of tumor cells). In the study, NED was associated with a shorter radiographic PFS in both the abiraterone (15.9 vs. 19.5 months, *p* = 0.010) and docetaxel cohorts (8.4 vs. 20.4 months, *p* = 0.016). Interestingly, the authors also noted that patients’ survival outcomes worsened as the NED proportion increased [48]. To our knowledge, our study represents the first analysis to evaluate the influence of NED on the efficacy of ARPIs in a European population. Notably, we explored not only the impact of NED in first-line treatment but also its effects in subsequent lines of therapy for mCRPC.

Notably, over 60% of PC with NED exhibit spatial intratumor heterogeneity with areas of cells with neuroendocrine differentiation intermixed with areas of acinar cells without NED.

Subsequently, we examined if the coexistence of these two cell subpopulations impacts ARPI efficacy by dividing the NED population based on the neuroendocrine component proportion.

Given the lack of an established and validated cutoff in the literature, we decided to use a limit of 50% neuroendocrine cells to differentiate between a high and low neuroendocrine component.

Remarkably, a worsening of PFS was observed among patients exhibiting high NED compared to those with low NED, both within the overall population and the pre-DOCETAXEL subgroup but not in the post-DOCETAXEL subgroup.

Consistently, Xu and colleagues also reported better outcomes in mCRPC patients with a limited proportion of neuroendocrine cells treated with first-line Abiraterone [48].

One potential explanation of these findings is that initially, ARPIs retain relative effectiveness, primarily due to their impact on acinar differentiating clones. However, during mCRPC progression, the selective pressure of therapy may have induced the expansion of androgen-independent cellular clones such as neuroendocrine tumor cells [53].

We can presume that following clonal selection, the neuroendocrine component could become predominant, explaining the important ARPI resistance shown in the post-DOCETAXEL setting and in mCRPC patients with high NED [48].

In summary, the clonal selection and the high aggressiveness of ARPI-resistant neuroendocrine cells lead to both shorter progression times on ARPIs and worse overall survival in patients treated post-docetaxel. This effect is less pronounced in earlier disease stages, where the deleterious effect of the neuroendocrine cells is partly attenuated by the acinar component.

ARPI use is increasing in earlier disease settings (e.g., non-metastatic CRPC [54] and biochemical relapse [55]). Preliminary findings indicate that NED evaluation at diagnosis refines the characterization of the risk of progression and could orient clinicians towards closer follow-up or tailored therapeutic approaches [56].

Finally, PC with NED should be considered a dynamic and heterogeneous disease, characterized by the coexistence of both areas of acinar cells that remain still sensitive to ARPIs, and areas of neuroendocrine tumor cells, that are highly resistant to ARPI therapy. In this biological context, future studies should evaluate the association of ARPIs with other treatments also targeting the neuroendocrine component [35,40,43].

Recently, numerous small phase I/II clinical trials have evaluated promising new therapeutic strategies against neuroendocrine prostate cancer and AVPCs. These include the combination of immunotherapeutic drugs (NCT03910660) [57], the association of chemotherapy and immunotherapy (NCT03582475) [58] or PARP inhibitors (NCT03263650) [44], and the use of bispecific T-cell engager anti-DLL3/CD3 (NCT04702737, NCT04471727) [45,59].

However, none of these studies are evaluating such strategies in combination with ARPIs.

Our represents the first analysis in a European cohort describing the negative prognostic impact of NED in mCRPC patients treated with ARPIs. However, we acknowledge several limitations.

Our study is single-center and presents selection bias; in addition, the retrospective nature of this analysis needs to be validated.

In most cases, tumor tissue for NED analysis has been collected at diagnosis limiting our ability to definitively confirm the hypothesis that clonal selection drives the worsening of outcomes in Docetaxel pre-treated patients. Furthermore, this precludes the evaluation of the impact of ARPI therapy on inducing neuroendocrine trans-differentiation in acinar cells. However, these objectives were beyond the scope of our retrospective study, and future prospective studies are required to verify these hypotheses generated by our findings.

In addition, not including a prospective re-evaluation of the neuroendocrine component (re-biopsies or serum biomarkers), this study cannot directly compare different diagnostic methods. Tumor re-sampling is useful for recharacterizing disease during its progression. However, the finding of suitable sites for re-biopsy is challenging in prostate cancer for both locoregional treatments on primary tumors and the tendency of prostate cancer to give bone metastasis, which are often technically less accessible sites for biopsy.

Therefore, the identification of tissue biomarkers at the time of initial diagnosis could be extremely useful in patients with prostate cancer.

Despite these limitations, our findings support the assessment of NED at the time of initial diagnosis of PC to predict patient prognosis in order to assist clinicians in selecting the most appropriate treatment for mCRPC patients.

## 5. Conclusions

The present study investigated the role of NED, assessed in tumor tissue, as a predictor of ARPI efficacy. Our findings revealed unfavorable clinical outcomes in patients with mCRPC with NED when treated with Enzalutamide or Abiraterone. These results underscore the importance of assessing NED and its proportion in prostate cancer, potentially assisting clinicians in predicting patient outcomes and optimizing treatment strategies.

## Figures and Tables

**Table 1:** Baseline treatment characteristics in overall population and comparison of baseline characteristics between metastatic castration resistance prostate cancer (mCRPC) with or without neuroendocrine differentiation (NED). Radiotherapy (RT); International Society of Urological Pathology (ISUP); prostate-specific antigen (PSA); hemoglobin (Hb); alkaline phosphatase (ALP); lactic dehydrogenase (LDH).

Baseline Characteristics	Overall Population	mCRPC with NED	mCRPC w/o NED	*p*
Number of patients	327	32	295	
Median age at diagnosis (range)	66.0 (47–87)	66.2 (47–86)	65.9 (87–47)	0.911
Age at diagnosis > 70 y (%)	108 (33.2)	11 (34.40)	97 (32.80)	0.926
Prostatectomy (%)	123 (37.7)	8 (25)	115 (39.1)	0.118
Prostate RT (%)	92 (28.1)	9 (28.1)	83 (28.1)	0.999
M1 at diagnosis (%)	153 (46.8)	22 (68.8)	131 (44.4)	0.014
ISUP GRADE (%)				
1–3	96 (34.3)	6 (19.4)	90 (36.1)	0.072
4–5	184 (65.7)	26 (80.6)	158 (63.9)	0.072
Neuroendocrine proportion (%)				
1–10%	17 (5.2)	17 (53)	NA	
10–50%	3 (1)	3 (9.5)	NA	
>50%	12 (3.7)	12 (37.5)	NA	
CHARTEED (%)	32 (9.8)	4 (12.5)	28 (9.5)	0.1
Previous-DOCETAXEL for mCRPC (%)	128 (39.2)	18 (56.3)	110 (37.3)	0.037
Drugs (%)				
Abiraterone	179 (54.7)	19 (59.4)	160 (54.2)	0.579
Enzalutamide	148 (45.3)	13 (40.6)	135 (45.8)	0.579
ECOG (%)				
0–1	313 (95.7)	30 (93.7)	283 (96)	0.784
2–3	14 (4)	2 (6.3)	12 (4)	0.784
Median PSA (min–max)	29.1 (0.1–5500)	29.5 (0.1–887)	24 (0.1–5500)	0.593
PSA > 50 ng/mL	126 (38.5)	14 (43.8)	112 (38)	0.481
Median Hb (min–max)	12.8 (6.8–17.9)	12.5 (6.8–14.4)	12.8 (7.6–17.9)	0.058
Hb > 12 g/L	222 (67.9)	20 (62.5)	202 (68.5)	0.067
Median ALP (min–max)	91.5 (35–1825)	100 (40–1825)	91 (35–1091)	0.383
ALP > 160 U/L	68 (20.8)	9 (28.1)	59 (20.1)	0.546
Median LDH (min–max)	235.5 (120–2273)	240 (156–1829)	235 (120–2273)	0.703
LDH > 240 U/L	125 (38.2)	12 (37.5)	113 (38.3)	0.616
Symptoms (%)	111 (33.9)	16 (50.6)	95 (32.2)	0.125
Use of opioid (%)	93 (28.4)	11 (34.3)	82 (27.9)	0.375
Metastatic site				
Bone metastasis (%)	262 (80.4)	27 (84.4)	235 (79.7)	0.321
Lymph nodes metastasis (%)	200 (61.2)	22 (68.8)	178 (60.3)	0.354
Visceral metastasis (%)	39 (12.1)	9 (28.1)	30 (10.3)	0.003
Liver metastasis (%)	13 (4)	7 (24.1)	6 (2)	0.001

**Table 2:** Treatment outcomes in patients with metastatic castration resistance prostate cancer (mCRPC) with neuroendocrine differentiation (NED) or without NED in overall cohort, pre- and post-DOCETAXEL setting. Prostate-specific antigen (PSA); progressive disease (PD); stable disease (SD); partial response (PR); complete response (CR); population (POP).

Treatment Outcomes	mCRPC with NED	mCRPC w/o NED	Odds-Ratio/Hazard-Ratio	*p*
**Best PSA response PD**				
Overall POP (%)	43.80	20.10	OR 3.09 [IC95 1.45–6.61]	0.002
Pre-DOCETAXEL (%)	28.60	15.60	OR 2.16 [IC95 0.63–7.36]	0.21
Post-DOCETAXEL (%)	55.60	28.40	OR 3.15 [IC95 1.23–8.83]	0.024
**PSA response > 50%**				
Overall POP (%)	46.90	67.50	OR 0.42 [IC95 0.20–0.89]	0.02
Pre-DOCETAXEL (%)	64.30	73.70	OR 0.64 [IC95 0.20–2.0]	0.422
Post-DOCETAXEL (%)	33.30	55.80	OR 0.40 [IC95 0.14–1.14]	0.08
**DCR (SD + PR + CR)**				
Overall POP (%)	24.10	62.10	OR 0.20 [IC95 0.47–0.1]	0.001
Pre-DOCETAXEL (%)	38.50	66.50	OR 0.32 [IC95 1.02–0.1]	0.067
Post-DOCETAXEL (%)	11.80	53.20	OR 0.12 [IC95 0.03–0.55]	0.02
**Median PFS (months)**				
Overall POP (%)	4.38	11.48	HR 2.51 [IC95 1.71–3.68]	0.01
Pre-DOCETAXEL (%)	8.49	14.93	HR 2.11 [IC95 1.16–3.82]	0.012
Post-DOCETAXEL (%)	4.01	7.47	HR 2.43 [IC95 1.45–4.05]	0.01
**Median OS (months)**				
Overall POP (%)	17.3	23.1	HR 1.37 [IC95 0.92–2.04]	0.122
Pre-DOCETAXEL (%)	43.1	29.2	HR 0.88 [IC95 0.45–1.75]	0.732
Post-DOCETAXEL (%)	12.53	18.03	HR 1.86 [IC 95 1.11–3.10]	0.016

## Data Availability

Data are contained within the article.

## Supplementary Materials

The following supporting information can be downloaded at: https://www.mdpi.com/article/10.3390/cells13161396/s1, Table S1: Treatment outcomes of overall population. Table S2: Univariate analysis for PFS in pre- and post-DOCETAXEL groups. Table S3: Univariate analysis for OS in pre- and post-DOCETAXEL groups; Table S4: Multivariate analysis for PFS and OS; Figure S1: Kaplan–Meier curves comparing PFS of patients with mCRPC with low NED (1–49% of tumor cells with NED) vs. mCRPC with high NED (≥50% of tumor cells with NED) NED vs. mCRPC without NED treated with Enzalutamide or Abiraterone. Figure S2A,B: Forest plot showing NED impact in different subgroups of patients on radiographic PFS (r-PFS).
