# Supplementary material for: Impact of Neuroendocrine Differentiation (NED) on Enzalutamide and Abiraterone Efficacy in Metastatic Castration-Resistant Prostate Cancer (mCRPC): A Retrospective Analysis
Source: Cells. 2024 Aug 22;13(16):1396. doi: 10.3390/cells13161396 (PMC11352349; doi:10.3390/cells13161396)
Supplement: Supplementary file 1 [file cells-13-01396-s001.zip › cells-3121340-supplementary.pdf]

Article

Impact of Neuroendocrine Differentiation (NED) on Enzalutamide and Abiraterone Efficacy in Metastatic Castration-Resistant Prostate Cancer (mCRPC): A Retrospective Analysis

Giovanni Farinea [1,*], Mariangela Calabrese [1], Federica Carfì [1], Isabella Saporita [1], Stefano Poletto [1], Marco Donatello Delcuratolo [1], Fabio Turco [2], Marco Audisio [1], Francesco Rosario Di Stefano [1], Marcello Tucci [3] and Consuelo Buttigliero [1]

[1] Department of Oncology, San Luigi Gonzaga University Hospital, University of Turin, 10043 Orbassano, Italy; mariangela.calabrese@unito.it (M.C.); federicamaria.carfi@unito.it (F.C.); isabella.saporita@unito.it (I.S.); stefano.poletto@unito.it (S.P.); donatello.m.delcuratolo@gmail.com (M.D.D.); marco.audisio@unito.it (M.A.); rosario-distefano@virgilio.it (F.R.D.S.); consuelo.buttigliero@unito.it (C.B.)
[2] Oncology Institute of Southern Switzerland, 6500 Bellinzona, Switzerland; fabio.turco@eoc.ch
[3] Department of Medical Oncology, Cardinal Massaia Hospital, 14100 Asti, Italy; marcello.tucci@gmail.com
* Correspondence: giovanni.farinea@unito.it

Citation: Farinea, G.; Calabrese, M.; Carfì, F.; Saporita, I.; Poletto, S.; Delcuratolo, M.D.; Turco, F.; Audisio, M.; Di Stefano, F.R.; Tucci, M.; et al. Impact of Neuroendocrine Differentiation (NED) on Enzalutamide and Abiraterone Efficacy in Metastatic Castration-Resistant Prostate Cancer (mCRPC): A Retrospective Analysis. *Cells* **2024**, *13*, x. https://doi.org/10.3390/xxxxx

Academic Editor: Alexander E. Kalyuzhny

Received: 8 July 2024
Revised: 1 August 2024
Accepted: 10 August 2024
Published: 22 August 2024

Copyright: © 2024 by the authors. Submitted for possible open access publication under the terms and conditions of the Creative Commons Attribution (CC BY) license (https://creativecommons.org/licenses/by/4.0/).

SUPPLEMENTARY MATERIALS

Outcomes	Overall population	pre-DOCETAXEL	post-DOCETAXEL
**Best PSA response (%)**			
PD	69 (22.5)	32 (16.6)	37 (32.7)
0-50%	37 (12.1)	20 (10.4)	17 (15)
>50%	200 (65.4)	141 (73.1)	59 (52.2)
**Best radiological response (%)**			
PD	110 (42.1)	59 (35.3)	51 (54.3)
SD	105 (40.2)	71 (42.5)	34 (36.2)
PR	36 (13.8)	27 (16.2)	9 (9.6)
CR	10 (3.8)	10 (6)	0 (0)
DCR (%)	151 (57.9)	108 (64)	43 (45)
**Median PFS (month, IC95)**	11.02 (9.24 – 12.80)	14.507 (12.44 – 16.56)	6.382 (4.97-7.80)
**Median OS (month, IC95)**	21.65 (18.64 – 24.65)	29.276 (23.39-35.16)	17.5 (14.19-20.82)

Table S1: Treatment outcomes of metastatic castration resistant prostate cancer (mCRPC) patients treated with Enzalutamide or Abiraterone in overall population, in pre-DOCETAXEL group or in post- DOCETAXEL group. progressive disease (PD); Stable disease (SD); Partial response (PR); Complete response (CR); Progression-free survival (PFS); Overall survival (OS)

Univariate analysis PFS pre-DOCETAXEL	Hazard Ratio	CI (inferior limit)	CI (superior limit)	p
Age > 70y	1.769	1.288	2.429	0.001
ISUP 4-5	1.383	0.959	1.995	0.083
Gleason > 8	1.137	0.907	1.426	0.266
M1	1.319	0.965	1.804	0.083
Prostatectomy	0.687	0.492	0.959	0.027
Radiotherapy	0.744	0.522	1.06	0.101
CHARTEED (+)	0.874	0.519	1.47	0.611
Symptoms (+)	1.448	1.067	1.966	0.018
Opioid use (+)	1.136	0.882	1.463	0.323
ECOG ≥ 2	1.276	0.705	2.306	0.421
Bone Metastases (+)	1.454	0.988	2.14	0.058
Visceral metastases (+)	1.475	0.834	2.609	0.181
PSA >50 ng/ml	2	1.52	2.632	0.001
Hb >12 g/dl	0.666	0.436	1.015	0.059
ALP >160 UI/L	1.408	1.146	1.729	0.001
LDH >240 UI/L	1.355	1.106	1.659	0.003
mCRPC with NED	**2.138**	**1.178**	**3.879**	**0.012**

Table S2A: Univariate analysis for progression-free survival (PFS) in pre-DOCETAXEL group. International Society of Urological Pathology (ISUP); Prostate specific antigen (PSA); Hemoglobin (Hb); Alkaline Phosphatase (ALP); Lactic dehydrogenase (LDH); metastatic castration resistant prostate cancer (mCRPC); Neuroendocrine differentiation (NED).

Univariate analysis PFS post-DOCETAXEL	Hazard Ratio	CI (inferior limit)	CI (superior limit)	p
Age > 70y	1.894	1.179	3.043	0.008
ISUP 4-5	0.939	0.63	1.4	0.757
Gleason > 8	1.069	0.835	1.369	0.595
M1	1.269	0.887	1.816	0.193
Prostatectomy	0.919	0.64	1.32	0.649
Radiotherapy	1.026	0.692	1.522	0.898
CHARTEED (+)	0.918	0.754	1.118	0.396
Symptoms (+)	0.981	0.782	1.23	0.867
Opioid use (+)	1.004	0.793	1.272	0.971
ECOG ≥ 2	0.796	0.623	1.018	0.069
Bone Metastases (+)	1.267	0.783	2.049	0.335
Visceral metastases (+)	1.986	1.255	3.145	0.003
PSA >50 ng/ml	1.026	0.78	1.351	0.852
Hb >12 g/dl	0.67	0.509	0.883	0.004
ALP >160 UI/L	0.978	0.792	1.206	0.832
LDH >240 UI/L	1.07	0.849	1.349	0.564
mCRPC with NED	**2.427**	**1.454**	**4.053**	**0.001**

Table S2B: Univariate analysis for progression-free survival (PFS) in post-DOCETAXEL group. International Society of Urological Pathology (ISUP); Prostate specific antigen (PSA); Hemoglobin (Hb); Alkaline Phosphatase (ALP); Lactic dehydrogenase (LDH); metastatic castration resistant prostate cancer (mCRPC); Neuroendocrine differentiation (NED).

Univariate analysis OS pre-DOCETAXEL	Hazard Ratio	CI (inferior limit)	CI (superior limit)	p
Age > 70y	2.187	1.547	3.092	0.001
ISUP 4-5	1.28	0.856	1.913	0.229
Gleason > 8	1.284	0.999	1.651	0.051
M1	1.441	1.023	2.029	0.037
Prostatectomy	0.521	0.354	0.767	0.001
Radiotherapy	0.712	0.484	1.046	0.083
CHARTEED (+)	1.012	0.581	1.763	0.967
Symptoms (+)	1.399	1.004	1.949	0.047
Opioid use (+)	1.18	0.897	1.552	0.236
ECOG ≥ 2	1.643	0.947	2.85	0.077
Bone Metastases (+)	1.228	0.804	1.874	0.342
Visceral metastases (+)	1.463	0.806	2.654	0.211
PSA >50 ng/ml	1.759	1.342	2.306	0.001
Hb >12 g/dl	0.62	0.413	0.929	0.021
ALP >160 UI/L	1.231	0.985	1.537	0.068
LDH >240 UI/L	1.211	0.973	1.506	0.086
mCRPC with NED	**0.892**	**0.452**	**1.759**	**0.741**

Table S3A: Univariate analysis for Overall survival (OS) in pre-DOCETAXEL group. International Society of Urological Pathology (ISUP); Prostate specific antigen (PSA); Hemoglobin (Hb); Alkaline Phosphatase (ALP); Lactic dehydrogenase (LDH); metastatic castration resistant prostate cancer (mCRPC); Neuroendocrine differentiation (NED).

Univariate analysis OS post-DOCETAXEL	Hazard Ratio	CI (inferior limit)	CI (superior limit)	p
Age > 70y	1.951	1.211	3.142	0.006
ISUP 4-5	1.135	0.757	1.701	0.539
Gleason > 8	0.968	0.763	1.227	0.787
M1	1.238	0.865	1.771	0.243
Prostatectomy	0.931	0.647	1.338	0.699
Radiotherapy	1.144	0.773	1.692	0.502
CHARTEED (+)	0.705	0.572	0.869	0.001
symptoms (+)	1.002	0.799	1.257	0.988
Opioid use (+)	0.964	0.76	1.223	0.764
ECOG ≥ 2	0.849	0.666	1.082	0.185
Bone Metastases (+)	1.486	0.923	2.394	0.103
Visceral metastases (+)	2.18	1.382	3.44	0.001
PSA >50 ng/ml	1.256	0.948	1.666	0.113
Hb >12 g/dl	0.657	0.495	0.871	0.003
ALP >160 UI/L	1.008	0.821	1.237	0.942
LDH >240 UI/L	1.087	0.866	1.365	0.472
mCRPC with NED	**1.86**	**1.115**	**3.101**	**0.017**

Table S3B: Univariate analysis for Overall survival (OS) in post-DOCETAXEL group. International Society of Urological Pathology (ISUP); Prostate specific antigen (PSA); Hemoglobin (Hb); Alkaline Phosphatase (ALP); Lactic dehydrogenase (LDH); metastatic castration resistant prostate cancer (mCRPC); Neuroendocrine differentiation (NED).

Multivariate analysis PFS	Hazard Ratio	CI (inferior limit)	CI (superior limit)	p
Age > 70y	1.769	1.299	2.409	0.001
ISUP 4-5	0.941	0.697	1.271	0.693
M1	0.889	0.607	1.303	0.547
Prostatectomy	0.846	0.602	1.188	0.334
Prostate RT	0.936	0.653	1.342	0.718
CHAARTED	1.015	0.801	1.286	0.903
PSA>50 ng/ml	1.403	1.071	1.837	0.014
Hb > 12 g/L	0.668	0.498	0.896	0.007
FAL>160 U/L	1.166	0.899	1.513	0.247
LDH>240 U/L	1.337	1.041	1.716	0.023
Bone metastases	1.596	1.096	2.324	0.015
Visceral metastases	1.523	0.981	2.363	0.061
Previous-DOCETAXEL CRPC	1.678	1.145	2.458	0.008
PS ≥2	0.611	0.424	0.882	0.008
Opioid use	0.919	0.707	1.195	0.53
symptoms	1.439	1.054	1.965	0.022
**mCRPC with NED**	**2.134**	**1.344**	**3.388**	**0.001**

Table S4A: Multivariate analysis for progression free survival (PFS). International Society of Urological Pathology (ISUP); Prostate specific antigen (PSA); Hemoglobin (Hb); Alkaline Phosphatase (ALP); Lactic dehydrogenase (LDH); metastatic castration resistant prostate cancer (mCRPC); Neuroendocrine differentiation (NED).

Table S4B: Multivariate analysis for overall survival (OS). International Society of Urological Pathology (ISUP); Prostate specific antigen (PSA); Hemoglobin (Hb); Alkaline Phosphatase (ALP); Lactic dehydrogenase (LDH); metastatic castration resistant prostate cancer (mCRPC); Neuroendocrine differentiation (NED).

**Multivariate analysis OS**	**Hazard Ratio**	**CI (inferior limit)**	**CI (superior limit)**	**p**
Age > 70y	1.833	1.316	2.555	0.001
ISUP 4-5	1.002	0.726	1.382	0.992
M1	1.013	0.67	1.533	0.95
Prostatectomy	0.763	0.526	1.106	0.153
Prostate RT	1.007	0.68	1.49	0.973
CHAARTED	0.786	0.612	1.009	0.059
PSA>50 ng/ml	1.297	0.994	1.692	0.056
Hb > 12 g/L	0.64	0.472	0.867	0.004
FAL>160 U/L	1.179	0.887	1.566	0.256
LDH>240 U/L	1.283	0.967	1.703	0.084
Bone metastases	1.384	0.938	2.043	0.102
Visceral metastases	1.95	1.238	3.072	0.004
Previous-DOCETAXEL CRPC	2.188	1.48	3.235	0.001
PS ≥2	0.711	0.477	1.06	0.094
Opioid use	0.901	0.677	1.2	0.476
symptoms	1.405	1.008	1.959	0.045
**mCRPC with NED**	**2.134**	**1.344**	**3.388**	**0.001**

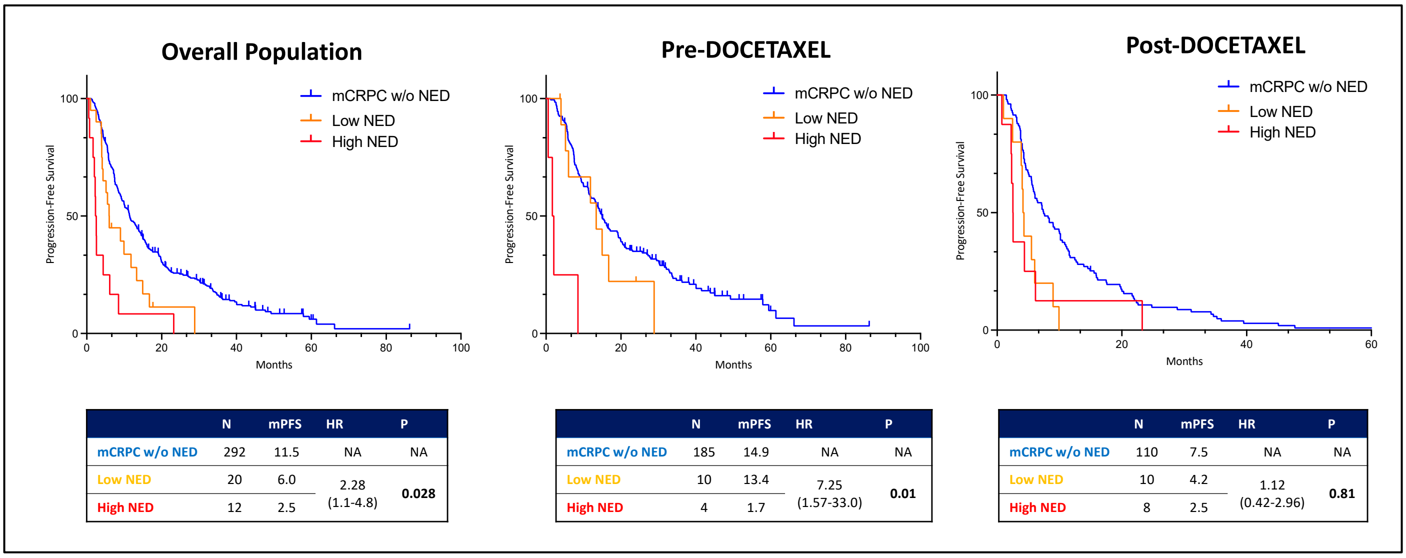

Figure S1. Kaplan-Meier curves comparing progression pre-survival (PFS) of patients with metastatic castration resistant prostate cancer (mCRPC) with low neuroendocrine differentiation (NED) (1-49% of tumor cells with NED) vs mCRPC with high NED (≥50% of tumor cells with NED) NED vs mCRPC without NED treated with Enzalutamide or Abiraterone. mCRPC with diffuse NED treated with ARPIs showed worse PFS compared with mCRPC with focal NED in overall population and in patients treated with ARPIs in pre-DOCETAXETAL setting.

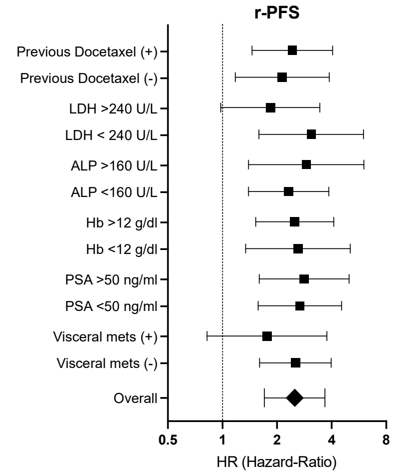

	r-PFS HR (CI min – max)	P (*)	mPFS NE (CI min - max)	mPFS no-NE (CI min – max)	N	P
**Prev. DOCETAXEL (+)**	2.138 (1.178 - 3.879)	0.751	8.48 (0.00 - 18.06)	14.93 (12.75 - 17.11)	199	0.012
**Prev. DOCETAXEL (-)**	2.427 (1.454 - 4.053)		4.01 (3.46 - 4.56)	7.46 (5.07 - 9.86)	128	0.001
**LDH >240 (U/L)**	3.087 (1.59 - 5.993)	0.271	5.16 (3.02 - 7.29)	19.18 (15.68 - 22.67)	140	0.001
**LDH < 240 (U/L)**	1.836 (0.979 - 3.444)		4.38 (0.53 - 8.23)	8.19 (5.62 - 10.75)	60	0.058
**ALP >160 (U/L)**	2.317 (1.392 - 3.856)	0.622	5.99 (4.58 - 7.40)	14.97 (12.91 - 17.02)	209	0.001
**ALP <160 (U/L)**	2.899 (1.393 - 6.034)		2.53 (1.98 - 3.08)	7.17 (5.36 - 8.97)	68	0.004
**Hb >12 g/dl**	2.707 (1.342 - 5.066)	0.921	2.34 (1.42 - 3.25)	6.09 (4.04 - 8.14)	68	0.005
**Hb < 12 g/dl**	2.607 (1.527 - 4.108)		5.99 (4.10 - 7.88)	14.8 (12.69 - 16.90)	222	<0.001
**PSA > 50 ng/ml**	2.669 (1.571 - 4.534)	0.887	8.49 (2.71 -14.28)	16.51 (12.97 - 20.04)	190	<0.001
**PSA < 50 ng/ml**	2.824 (1.595 - 4.998)		2.57 (0.00 - 5.23)	7.20 (5.63 - 8.76)	126	<0.001
**Visceral mets (+)**	1.761 (0.823 - 3.771)	0.426	3.88 (0.06 - 7.71)	6.09 (3.83 - 8.34)	39	0.145
**Visceral mets (-)**	2.524 (1.602 - 3.979)		5.16 (2.53 - 7.79)	12.89 (10.77 - 15.01)	283	<0.001
**Overall**	2.505 (1.705 - 3.68)		4.40 (2.46 - 6.30)	11.48 (9.51 - 13.44)	327	<0.001

Figure S2A. Forest plot showing neuroendocrine differentiation (NED) impact in different subgroups of patients on radiographic progression free survival (r-PFS). The negative impact of neuroendocrine differentiation on androgen receptor pathway inhibitors (ARPIs) efficacy was noted across all subgroups. **(*) Interaction test**

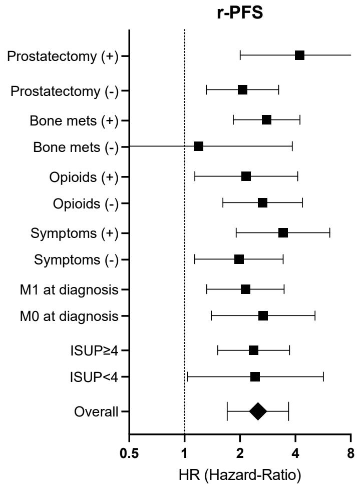

	r-PFS HR (CI min – max)	P (*)	mPFS NE (CI min – max)	mPFS no-NE (CI min – max)	N	P
**Prostatectomy (+)**	4.231 (2.007 - 8.919)	0.107	2.5 (0.22- 4.77)	13.84 (10.27 - 17.42)	123	<0.001
**Prostatectomy (-)**	2.068 (1.315 - 3.25)		5.16 (3.15 - 7.17)	10.16 (8.17 - 12.15)	203	0.002
**Bone mets (+)**	2.795 (1.842 - 4.243)	0.179	4.38 (2.42 - 6.32)	11.11 (9.58 - 12.65)	262	<0.001
**Bone mets (-)**	1.189 (0.367 - 3.851)		14.93 (2.44 - 27.4)	15.92 (11.61 - 20.22)	64	0.773
**Opioids (+)**	2.165 (1.137 - 4.121)	0.622	2.53 (0.76 - 4.29)	7.47 (3.75 - 11.18)	86	0.019
**Opioids (-)**	2.655 (1.616 - 4.362)		5.16 (2.64 - 7.68)	13.06 (10.40 - 15.71)	214	<0.001
**Symptoms (+)**	3.428 (1.91 - 6.15)	0.180	3.88 (0.66 - 7.10)	8.03 (6.57 - 9.48)	111	<0.001
**Symptoms (-)**	1.975 (1.136 - 3.434)		5.53 (0.0 - 14.94)	14.24 (11.63 - 16.84)	198	0.016
**M1 at diagnosis**	2.142 (1.32 - 3.477)	0.589	5.16 (2.88 - 7.43)	9.21 (7.09 - 11.32)	153	0.002
**M0 at diagnosis**	2.677 (1.399 - 5.124)		4.14 (3.63 - 4.65)	13.42 (10.51 - 16.32)	174	0.003
**ISUP≥4**	2.372 (1.514 - 3.716)	0.963	6.15 (0.92 - 11.37)	12.83 (8.43 - 17.22)	184	<0.001
**ISUP<4**	2.426 (1.037 - 5.678)		4.38 (2.50 - 6.25)	11.28 (8.23 - 14.32)	96	0.041
**Overall**	2.505 (1.705 - 3.68)		4.37 (2.46 - 6.29)	11.48 (9.51 - 13.44)	327	<0.001

Figure S2B. Forest plot showing neuroendocrine differentiation (NED) impact in different subgroups of patients on radiographic progression free survival (r-PFS). The negative impact of neuroendocrine differentiation on androgen receptor pathway inhibitors (ARPIs) efficacy was noted across all subgroups. **(*) Interaction test**
